# Situational Analysis for Complex Systems: Methodological Development in Public Health Research

**DOI:** 10.3934/publichealth.2016.1.94

**Published:** 2016-03-10

**Authors:** Wanda Martin, Bernie Pauly, Marjorie MacDonald

**Affiliations:** 1 College of Nursing, University of Saskatchewan, Saskatoon, SK, Canada; 2 School of Nursing, University of Victoria, Victoria, BC, Canada

**Keywords:** Situational Analysis, methodological development: complex adaptive systems, public health, implementation

## Abstract

Public health systems have suffered infrastructure losses worldwide. Strengthening public health systems requires not only good policies and programs, but also development of new research methodologies to support public health systems renewal. Our research team considers public health systems to be complex adaptive systems and as such new methods are necessary to generate knowledge about the process of implementing public health programs and services. Within our program of research, we have employed situational analysis as a method for studying complex adaptive systems in four distinct research studies on public health program implementation. The purpose of this paper is to demonstrate the use of situational analysis as a method for studying complex systems and highlight the need for further methodological development.

## 1. Introduction

There have been consistent calls for strengthening and renewing public health worldwide in an effort to improve population health and to reduce health inequities [1]–[3]. The implementation of effective public health programs and policies is critical to advance population health and promote health equity. Understanding the process of policy and program implementation is important for two main reasons: first, to ensure that it is, in fact, the public health programs that lead to population health outcomes, and second to identify the necessary organizational supports for implementation of public health interventions to inform public health practice. Studying public health programs in complex systems presents many research challenges demanding methodological development to generate knowledge about successful implementation of public health programs and services. Sharing examples of newer methodologies can advance thinking and contribute to application in various contexts. The purpose of this paper is to describe situational analysis as a newer research method for studying complex public health systems, using original research to illustrate the potential of this methodological approach for public health systems and services research.

MacDonald, as one of the team leads of the Research in Public Health Systems and Services Initiative (RePHSS) in British Columbia (BC), Canada, formerly called the Core Public Health Functions Research Initiative, identified studying the implementation and impact of public health programs and policies as a priority to advance population health and to promote health equity [4]–[6]. The implementation of public health programs and policies occurs within complex systems which Anderson, Crabtree, Steele and McDaniel [7] describe as organic, dynamic, and living social systems. Within a complex system, context is central to the implementation and impact of programs and policies. Pettigrew [8] defines context not only as circumstances in the environment, but as a nested arrangement of structures and processes shaped by those involved in the environment. According to Poland, Frohlich and Cargo [9] it is the social context that shapes how the phenomena are taken up, resisted, or modified. In fact, from a complexity perspective, context interacts with the intervention to produce outcomes, which then feed back into the system to change the context, which again interacts with the ongoing intervention to intensify, enhance, or change the outcomes. This reflects ecological complexity in reciprocal and iterative fashion.

Although many public health authors recognize and acknowledge the importance of context in understanding the workings of complex public health systems, there has been very little methodological development in the study of context [10]. Most research that reports on contextual influences on public health interventions draws on survey data to identify elements of context that correlate with outcomes, but provide little data that explain the causal processes and linkages between outcomes and context [11]. One way to study context is through mapping causal processes and working through the complexity of these relationships to gain insight into the conditions that trigger causal mechanisms to produce outcomes. Such linkages and relationships are often missed using traditional or conventional theories and methods.

Early in the process of developing a program of research in public health systems renewal, MacDonald recognized that traditional experimental designs with random selection of participants or manipulation of interventions, do not work well in real-world and complex public health settings [4] because context is often missed. Therefore, team members sought out emerging methods and research designs not typically utilized in public health research but that seemed suitable for studying context and mapping causal processes. The RePHSS team has explored various methods for mapping complexity, and have applied a range of methods in different research projects. One such method was Situational Analysis [12].

In this paper, we focus on the study lead by Martin (at the time a nursing doctoral student) and supervised by MacDonald and Pauly. We describe how we applied this research analysis method with a complex adaptive systems framework in British Columbia (BC), one Canadian province. In the study “Food Gone Foul?: Food Safety and Security Tensions” Martin [13] used Situational Analysis [12] to understand the context of the food system and the relationships among professionals in three areas: those working in the Food Safety Core Program, those involved with the Food Security Core Program, and people working in community food security. By community food security, we mean “... a situation in which all community residents obtain a safe, culturally acceptable, nutritionally adequate diet through a sustainable food system that maximizes community self-reliance and social justice” [14]. We obtained ethical approval for the study from the University of Victoria/Vancouver Health Authority Joint Ethics Review Board (J2011-23) and obtained written consent from participants.

We formulated research questions for Food Gone Foul through conversations with RePHSS knowledge users at the time that the health authorities were implementing the new Food Security Core Program. The questions were: (a) how are intersecting areas between food safety and food security negotiated, and (b) what are the facilitators and constraints to collaboration? Together, food safety and food security fit within, but are only part of, the larger and more complex food system and have multiple systems nested within them. Studying the relationships between these nested systems required a theoretical perspective with a focus on context, such as complex adaptive systems.

## 2. Complex adaptive systems

Complexity science is not one singular theory but consists of numerous theories and frameworks interpreted through the lenses of multiple disciplines [15]. One way to describe a complexity science worldview is complex adaptive systems. This worldview provides a vision of relational and dynamic life that is holistic, self-organizing, interconnected, nonlinear, and evolving [16]. Capra [17] describes complex adaptive systems as a study of problems in adaptive, self-organizing systems that are not explained by methods of traditional science. Holland [18] refers to complex adaptive systems as systems that maintain coherence under change. Essentially, complex adaptive systems are about understanding a set of principles that rule system behaviour such as nonlinearity and emergence [18]. Research using this worldview has a focus on the context involving study of relationship patterns, how relationships are sustained, and how outcomes emerge, with an emphasis on the whole and on synergy, rather than on individual parts [19].

Various principles are emphasized by different authors, but for the purpose of this research, Martin [13] drew on work by Anderson and colleagues [7] using case study design with a complexity science blueprint. Case study is itself a method to study complex systems, but Anderson and colleagues [7] refer to specific characteristics as a map or a blueprint that reflect complexity science concepts. According to these authors [7], there are five key characteristics of complex adaptive systems. The characteristics include: (1) agents who are people and processes (human and non-human actors) with the capacity to relay or exchange information; (2) nonlinear interconnections of agents that can be recognized as widespread patterns; (3) self-organization in which groups come together without external direction; (4) emergence or the development of system properties that are not characteristic of the individual parts; and (5) co-evolution in which the development of one system may be linked with the development of another outside system. Ideally, researchers can utilize these principles to recognize opportunities for studying context and implementation and for solving problems within the system. This thinking is highly relevant to public health because human health is a complex process operating through interactive systems [20]. Thus, there is a need to identify appropriate and relevant research methods to study problems in this field and contribute to the advancement of population health and health equity.

In developing Situational Analysis as a new approach to grounded theory analysis, Clarke [12] does not identify specifically the need to operationalize complexity science or complex adaptive systems as rationale for this new approach. However, she does recognize the opportunity to use the strength of the grounded theory process as developed by Glaser and Strauss [21], with less of a focus on the “basic social process” in favour of the social worlds/arenas/negotiations framework that comes from Strauss's pragmatist philosophy and symbolic interactionism [12]. Social worlds are those in which there are shared commitments and attitudes toward social organization within arenas [22]. This includes collective action and a shared dialectical approach that defines the social world [23]. Additionally, the ecological nature of the conditional matrix used in grounded theory, along with the emphasis of situational analysis on context, provides theoretical links between situational analysis and complexity science. In situational analysis, the social world and collective commitments and actions of participants within that world are the units of analysis. Complexity science is evident in situational analysis through these concepts of the social world where relationships and the context are an important source of meaning. It is the point of the analysis to uncover the nature of interconnections (both an ecological and a complexity concept) within and among the arenas of action. Complexity concepts of relationships, nonlinearity, and self-organization are part of understanding the social worlds and arenas because they provide an understanding of what is happening in that social world, and how the social world relates to and is different from others in the arena.

Below we provide a description of situational analysis as a methodology for studying complex adaptive systems with an original research project to study tensions in the implementation of food safety and food security core public health programs. Published works using situational analysis are limited and, therefore, few examples were available as a model of how best to report information that can be complex to understand and to describe. By describing study findings, we hope to illustrate how to report situational analysis of complex adaptive systems.

## 3. Situational analysis

Situational analysis is an approach to research using a grounded theorizing methodology to identify and describe social worlds and arenas of action and by representing complexity through mapmaking [12]. Clarke [12] has taken grounded theory beyond the more constructivist approach of Charmaz and Morse [24] to create a new process of analysis that is situation-centred (i.e., largely centred on context) and focused on a social worlds/arenas/negotiations framework [25]. Situational analysis is beneficial to open up the data by providing a comprehensive framework for considering multiple connections and relationships that can influence activities [12] – in this case, elements of the situation that influence implementation of core public health programs. Using explanatory maps, situational analysis provides unique visuals for understanding the phenomenon of interest and considerable potential for visual representation of data to aid in knowledge translation activities. Visual complexity, as a means to recognize patterns and to clarify complex systems, is part of a growing trend in social science [26]. It allows the reader to see how complex the situation is, while demonstrating how various parts, through interaction, influence outcomes.

Situational analysis provides a means to specify and map all the important human and nonhuman elements of a situation, emphasizing relationships, social worlds and discursive positions [12]. Specifically, the methodology for situational analysis involves substantive theorizing and story-telling through the use of maps with a goal of critical analysis to produce a possible ‘truth,’ or the underlying structure or mechanism of action [12]. Clarke [12] identifies three main types of maps to help understand the situation: 1) situational maps, 2) social worlds/arenas maps and 3) positional maps. The maps are central to the process of analysis and provide visual representations of the findings.

### 3.1 Situational maps

To create the maps, situational analysis begins as with most grounded theory studies: gathering data through interviews, documents, and other sources that contribute to illuminating the basic social problem [21]. Clarke [12] describes conventional grounded theory coding and memo writing [21] as part of the process to become familiar with the data and also indicates that partially coded but carefully read data is sufficient to begin. Our experience is that constant comparative analysis is appropriate, without the depth required of various levels of coding in the conventional grounded theory process. Clarke [12] describes the maps as analytical exercises to stimulate thinking and to free the researcher from “analytic paralysis” (p. 84) that can occur when simply reading over data and recording memos.

Situational maps aid in articulating arguments or assertions and other elements of a situation and the relationships among the elements [12]. This begins with a ‘messy map’ created by descriptively laying out all the human and nonhuman elements by asking ourselves: who and what are in this situation; who and what matters in this situation; and what elements make a difference in this situation [12]. These descriptors are randomly ‘dumped’ on a large sheet of paper so the team can focus on ideas and not be concerned with the structure at this point. For example, Martin [13] explored four cases in the situation of tensions between those working in food safety and in food security (community kitchens, farmer's markets, unpasteurized milk, and urban chickens). She identified unique aspects of each case in each corner of a large sheet of paper, with common elements in the centre. This map helps the analyst to frame the situation and consider what might be invisible, or a taken-for-granted aspect of the situation [12]. This map is not a final version of who or what is in the situation, but it is as a starting point to stimulate thinking and to start to recognize patterns and connections in the data.

Derived from messy maps are ordered maps (Table 1). Once the elements of what matters are down on paper, they are grouped or categorized to make sense for this topic area. Clarke [12] provides 12 suggested headings for the ordered map but encourages the researcher to use headings that make sense for the data to be understood. We describe these headings below. Headings help to identify and group the elements and actors, who are *individuals, groups, or non-human entities*. *Implicated silent elements/actors* are such things suggested by the data sources, but there is little direct information from individuals or groups in this category. For example, Food Secure Canada is not mentioned in the data, but is part of the way that other actors, such as the BC Food Systems Network, talk about food security.

The *discursive construction* category helps to tease out the story lines about what is happening in a situation. By discursive construction, we are referring to ideas, symbols, concepts, discourses, debates and cultural aspects of the situation that may have some significance in understanding the basic social problem [12]. We found that identifying elements in this category to be challenging to determine what we considered a human versus non-human actor. In a subsequent study, we decided that a discursive construction of human elements was not about the constructions of things articulated by people, but was more about how some people were being constructed by the informant.

Identification of political, *economic and sociocultural elements* assists the analysts in thinking through the broader context of the situation. It is important to consider how each of these elements may influence program implementation and collective action.

The temporal *elements* category refers to events that have influenced the situation over time or anything related to time, and the *spatial elements* refer to the way the distance and space affect the situation. Finally, there are *related discourses*, and *major issues or debates* found in the data that are important to consider for understanding the situation. Debates in the data are particularly important in developing positional maps, we describe below. The exercise of developing the ordered map helps to consider elements and discourses influencing the situation that we may otherwise overlook.

To produce the ordered map for the food system research, Martin [13] took all the elements and actors from the messy map, and positioned them under Clarke's headings. Thinking of the descriptions in terms of the categories stimulated identification of areas that were not otherwise obvious, particularly for a novice researcher. For example, discussions about community kitchens included data about the food-provisioning group “Food Not Bombs”. It was the inclusion of this group, as a collective human element, that highlighted their role in food provision, because that organization does not fit within the original conceptualization of community kitchens by the researcher. The importance of this group, who use food resourced from dumpsters or other questionable sources to feed street involved people, was made obvious when given an equal place on the ordered map as other aspects of the food safety/food security situation. This map was part of the analytical process to move from data on specific cases to develop and describe social worlds/arenas. Martin [13] placed the elements in the situation in these groupings as a way to organize her thinking and it was not intended to ‘box in’ concepts, but to provide a beginning framework for analysis. The situational maps have the freedom to grow and change and can inform theoretical sensitivity and drive further data collection.

**Table 1:** Ordered Map

**Individual human elements/actors** Animal Control and Bylaw officers Canadian Food Inspection Agency Inspectors City Planners Community Kitchen Leaders/facilitators Community Nutritionists Environmental Health Officers Farmers (Small Scale Producers) Food security coordinators Health Authority directors and managers Lawyers and Judges Market Coordinators and Vendors Michael Schmidt – Raw Milk Farmer Milk Shareholders Municipal councillors People at risk (pregnant, children, seniors, street involved) Public (Chicken owners, eaters)	**Nonhuman elements/actors** Animals – Chickens, Roosters, Cows and Goats Bacterial contaminants (listeria, E. coli, etc.) Public Health Act Farm Markets & Grocery Stores Food Safety Act FoodSafe Course Free range, free run and caged eggs HACCP Kitchens Laboratories MarketSafe Course Meat Inspection Regulations Mobile Slaughter Raw and pasteurized milk Social and Mainstream Media (Internet) Statistics and data
**Collective human elements/actors** BC Centre for Disease Control BC Farmers' Market Association BC Food Systems Network Canadian Food Inspection Agency Food Banks Food Not Bombs Fresh Choice Kitchens Health Authorities Ministry of Health Provincial food safety/food security working group Public Health Victoria Community Kitchens Network	**Implicated silent elements/actors** Agriculture Farm Land Antibiotics Avian Flu Canadian Institute of Public Health Inspectors Core Functions Framework Dietitians of Canada Food Secure Canada Industrial Food System Marketing Boards Ministry of Agriculture and Lands Municipal, Provincial, Federal Governments Underground economy (black market)
**Discursive constructions of individual/collective human actors** Fear of inspectors Food as a human right Food waste and how to get food to hungry people Healthy food system Understanding the science behind causes of foodborne illness Understanding where food comes from, or the connection b/w farmer and consumer (distancing)	**Discursive construction of nonhuman actors** Chronic disease prevention and healthy eating Climate Change affecting food production Local food movement and food miles Poor quality data on foodborne illnessSustainable farming (Pesticide use, Sewage sludge) Terrorist Duck (i.e. a wild animal spreading disease) Traceability of food
**Political/economic elements** Conservative government cuts to food inspection Food as a right & UN's Special Rapporteur's visit to Canada Protection of the public Quota system, Marketing Boards, and Trade	**Sociocultural/symbolic elements** Food skills Health equity Income level Food Labeling
**Temporal elements** BSE and Avian flu crisis Caged eggs changes in regulations Home on the Range and Michael Schmidt court cases Kildara Farm salad greens recall Maple Leaf foods listeria outbreak Right to food court case challenging backyard hens in Calgary	**Spatial elements** Distance between dairy farms and milk shareholders Distance between farms and abattoirs Farm Gate Sales Food imports and Local food Provincial boundaries for trade Rural and remote areas of BC
**Major issues/debates (usually contested)** Allowing urban chickens Can small-scale feed the population? Meat Inspection regulation changes Perceived risk and over-sanitization/dirt therapy Raw versus pasteurized milk Scale-appropriate regulations	**Related discourses (historical, narrative, and or visual)** 100 mile diet Egg refrigeration and washing Global hunger Melamine contamination in pet food and China's food safety system

### 3.2 Social worlds/arenas maps

**Figure 1:**
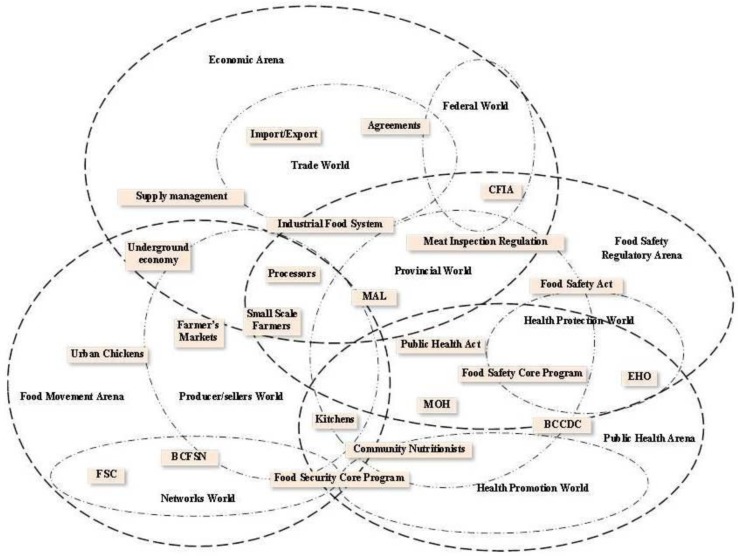
Social Worlds/Arenas Map

A second category of maps used to explore the situation is social worlds/arenas maps (Figure 1). This category locates social action or activity at the intermediate (meso) level of a situation and demonstrates where discourses are active [12]. For example, the ordered map shows a range from the micro to macro level of specific actors and elements in the situation while the social worlds/arenas map highlight groups, such as departments in health authorities, food security networks, and regulatory agencies. Arenas are “a field of action and interaction among a potentially wide variety of collective entities” [23] and includes actors such as organizations, social worlds, new social movements, ideologies, and technologies. The term “social worlds” comes from the work of Strauss. Clarke [23] describes these as universes of discourses in arenas, and they include collective action and shared commitments that define the social world. Mead [27] used the term “universes of discourses” to capture the idea that understanding a phenomena can occur through examining what is embedded in relationships. The meaning is made by working together for a common goal.

Specifically, Martin [13] was concerned with tensions expressed and experienced by actors working in food safety and food security and how those actors communicate and collaborate (or not). The questions asked of the data in creating this map were: what are the patterns of collective commitments of the actors and the salient social worlds; who is participating or who is not; why or why not are they participating; and what are the characteristics of the social world? [12]. The social worlds are fluid, as there is no true boundary between them, and they can overlap since people are often located in more than one social world at a time [12].

**Figure 2:**
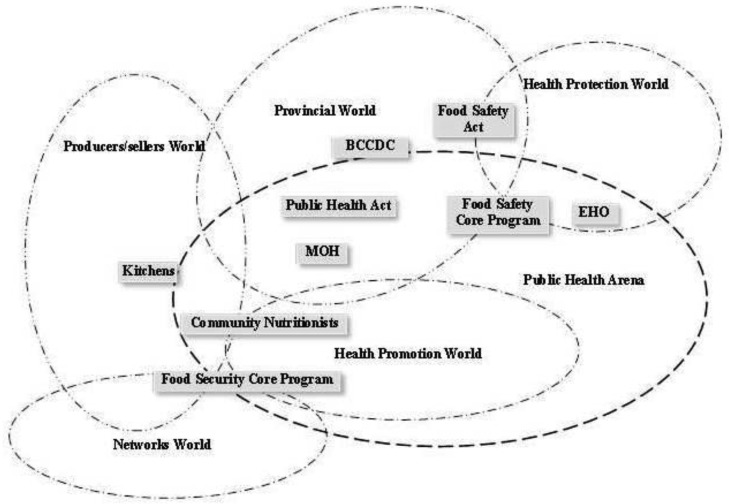
Public Health Arena

This map has four main arenas reflecting the tensions between food safety and food security, shown by the darker lines: food movement, public health, food safety regulatory, and economic. There are seven social worlds that are within and crossover arenas, shown by the lighter lines: networks, producers/sellers, health promotion, health protection, provincial, federal, and trade. Within that situation are groups of human and nonhuman actors that sit in or across social worlds and arenas shown in the boxes. In Figure 2, we isolate just the public health arena to illustrate the type of information derived from this map, focusing mainly on the health promotion and health protection worlds. While other worlds in the diagram cross into this arena, there is a broader discussion of those worlds in the dissertation[13], within the arena sections. One of the biggest challenges we experienced in doing situational analysis was writing the findings, which are non-linear, in a linear manner to draw a coherent picture for the reader.

Here we focus on one of the arenas to illustrate the complexity of the situation, and challenges in describing the context. From the theoretical perspective of complex adaptive systems, this map shows a holistic view or interconnecting worlds and actors. It is the patterns of relationships that placed these actors and worlds in the positions in which they are located on this map. In this situation, the public health arena (field of action) involves the provincial world, health promotion world, health protection world and a small piece of the producer/sellers and networks worlds. Each of these social worlds has commitments to activities that build their interest. Traditionally, public health actors who worked with food were involved with chronic disease prevention, healthy eating, and inspection of food premises that cook and serve food for sale. More recently, food security discourse and action in public health has grown, expanding on traditional public health work to include agriculture, environment, and food systems policy. In BC, this is partially due to the introduction of the food security core public health program. Promotion of healthy food consumption and access to food are part of the health promotion world. In this world, professionals such as community nutritionists, community health nurses, or community developers work with populations to identify solutions to local health needs. Community nutritionists are educated as dieticians and regulated under the College of Dieticians. Their nutrition training includes food safety, and they work closely with community members on food security activities such as food banks, food reclamation (reducing food waste), community gardens and kitchens, food policy, and Indigenous food systems. Community nutritionists have a strong interest in health equity.

The food security core program made explicit the scope of work necessary to support healthy communities [28]. It is important for public health to be involved in health promotion activities that directly tackle obvious issues of hunger and poor quality diets, but also to work in a systemic way, with vision to support producers and sellers. This includes reducing exploitation of farm workers in the agri-food system and working toward food justice for basic human needs [29]. The Food Security Model Core Program paper[28] does not explicitly name food justice as an objective, but does support the use of environmentally sustainable production and distribution methods, supporting the concept of food justice as described by Allen [29].

The health protection world consists mainly of EHOs as key actors, also referred to as health inspectors. They have four areas of work: air quality, land use as it affects the environment, water quality, and food safety. While land and water may affect food production areas, the work is not in food production but mainly in sewer systems and swimming pools. Food safety work of the EHO is primarily inspection of food premises (restaurants or temporary food markets) and food production facilities where food is prepared for the market place. Work of the EHO relies heavily on understanding and applying the Public Health Act [30] and the Safe Food for Canadians Act [31]. EHO education and training provides them with a certain worldview of owning power and authority that may appear antagonistic to small-scale producers.

The health promotion and health protection worlds sit in the public health arena but they do not overlap. The difference between these worlds is in the approach to the food situation. Dieticians in this study described their desire for people (usually those who are most at risk of food insecurity) to feel comfortable in dealing with them, while the EHO is seen in the role of enforcer and protector. These are two very different styles of working with people although both professions have the same goal of a safe and accessible food supply. At times, they get a very different response from people they serve. There is an approach to community that involves working collaboratively and in an egalitarian way with people (“doing with or power with”), and a more top-down approach that involves power and control over people (“doing to or power over”) [32]. Although a number of EHOs do approach their work in a community development-like way, people in this study talked about the EHO's reputation of enforcer and protector getting in the way of relationships with producers and sellers.

The distance in the diagram between EHO's and actors in the food movement arena represents the distance described by study participants. Only a small portion of informants in the food movement arena had an established relationship with an EHO. Those who did were less fearful of the outcomes of inspection. A seller at a farmer's market noted “She [the EHO] definitely is in control of us. I mean she's good to work with but she was pretty scary at first. So I guess that's how they establish authority.” Considered by some as “sanitary police,” EHOs generated a mixed reaction from their clients. Some people expressed fear of authority figures because of the power they have to affect the lives of people who they may see as non-compliant. Others see compliance with food safety regulations as good business. If the EHO is not approachable, there are fewer opportunities for the EHO to educate and support producers or sellers in safe practice. The center of the problem may be in the misfit of regulations for small-scale producers and the fact that the EHO has little power to modify regulations.

The public health arena is only one piece of the story describing this situation. The other arenas depict the situation from the economic perspective, the changes in the developing food movement, and the details of food safety regulation. Each arena has a story describing the situation. It is through these descriptions that we can begin to see patterns evolve, and the way that groups self-organize, as in the developing food movement. The focus on the relationships in situational analysis highlights the interconnected nature of complex adaptive systems.

### 3.3 Positional maps

**Figure 3:**
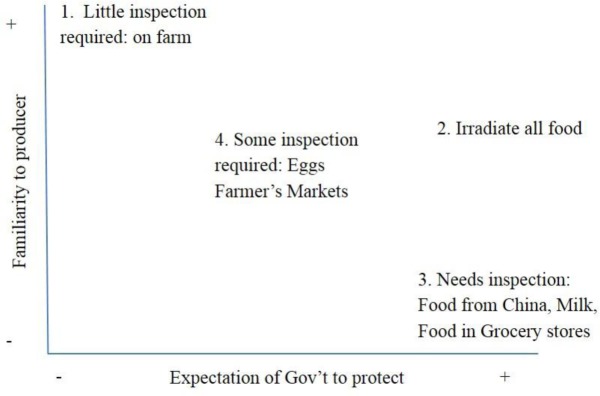
Positional Map

The final category of maps Clark [12] uses to chart data is the abstract positional map (Figure 3) which shows various positions within major discursive issues arising in the data. Positional maps highlight the range of positions on issues, not those associated with individuals, groups or institutions but positions in discourses as reflected in the data [12]. This type of mapping attempts to separate the politics of representation from the discourse and helps to identify the complexity of emerging behaviours [12]. That is to say, positional maps are higher order conceptualizations of positions in the data, not linked to individuals or groups. Clarke [12] argues that positional maps, which are free of associations with individuals or institutions, help the researcher to see situations better because they are representative of the larger picture, where there is a broader view. It is this holistic view that corresponds to the theoretical approach of complex adaptive systems.

As an example of how situational analysis is useful for illustrating complexity and context, we provide the positional map in Figure 3. This map is based on familiarity people have with the food producer and their expectations for government to protect the population from foodborne illness. The closer activities get to an anonymous market of producers and consumers, the greater the expectations for government to be involved food safety protection. The x-axis represents the expectations participants have of the government to provide protection, and ranges from no expectation (-) to full expectation (+). The y-axis represents the familiarity of participants with the producer, and ranges from no familiarity or being total strangers (-) to great familiarity (+).This positional map illustrates the idea that familiarity leads to trust, and trust reduces the need for costly investments in safety monitoring or enforcement institutions [33].

The positional map tells a story of its own. The first position in the data is ‘little inspection required' (1) with low public expectations for the government to protect on the x-axis and high degree of familiarity between producer and consumer on the y-axis. This position involves active participation in acquiring food from a friend who is a farmer. There is a high level of trust and appreciation for the work involved in growing food, and regulatory oversite may appear as an additional burden for farmers who the public see as already working hard to keep the farm viable. This position is also evident in discourse about the concept of ‘food sovereignty’ [34]. The emphasis is on local food production respecting the environment, farm workers, and local empowerment. By food sovereignty we mean a process of expanding democracy to regenerate local, autonomous, healthy and ecologically sound food systems that respect the right of people to decent working conditions and incomes [35], [36]. It is a food system apart from industrial agriculture.

The second position in the data on this map is to ‘Irradiate all food' (2), grounded on the high end of expectation for government to protect on the x-axis and mid-level degree of familiarity on the y-axis. The familiarity with the farmer is of no consequence for this position, because of the concept that all food is potentially high risk for foodborne illness. For a slight decrease in quality, society would gain a significant reduction in illness. This is a highly contested position by many local growers. There is a position, however, where many warrant a high degree of regulation because of the technological advances in the ways we can eradicate foodborne illness.

The third position on this map, ‘Needs inspection’ (3), is on the high end of expectation for government to protect on the x-axis and a low degree of familiarity on the y-axis. This would include imported food, especially from China due to high pesticide use and the use of melamine as a food additive. This also applies to food sold in grocery stores. This position favours regulations for large anonymous food systems and it not usually contested.

The final position for this map is number four: ‘some inspection required’ (4), which is centred between both axes. Eggs and farmers' markets are included in this position. Eggs are primarily from large-scale producers and go through a grading station, but there are also ungraded eggs for sale in smaller grocery stores and farmer's markets. Similarly, with farmer's markets, there were a number of patrons who were happy with the extent the government regulates them. A number of people felt more regulations would affect the vendors and, therefore, decrease the scale and quality of the markets. The story from this positional map is that the public expects a certain amount of regulatory oversight, but too much restriction is undesirable largely where there is the demand for local food.

The result with situational analysis is not a substantive theory as with grounded theory, but maps representing substantive theorizing that describes and explains the situation [12]. In Food Gone Foul, the story was about trust and relationships to allay fears of unsafe food, lack of food, and lack of control over the food supply for those who are seeking food sovereignty.

The social worlds/arenas maps are not as intuitive for relaying a story as one might find with a grounded theory diagram, but the maps are rich in demonstrating complexity. In writing on Food Gone Foul, Martin [13] displayed and described each of the four arenas separately, followed by describing the various position in the data. The challenge was to give a linear reading to a nonlinear situation, while retaining the iterative and complex nature of the relationships.

## 4. Situational analysis and complex adaptive systems

Situational Analysis is a relatively new method stemming from grounded theory that can be used to map context and complexity of situations. Clarke, however, did not develop the method specifically in relation to principles and properties of complex adaptive systems thinking. Applying Anderson and colleagues [7] blueprint for complex adaptive systems was a way to focus discussion in the Food Gone Foul study, but more work is needed in this area to recognize evidence of emergence, nonlinearity, self-organization, and co-evolution. Evidence of these traits may be more apparent in longitudinal studies, where the research can track changes to recognize the process of self-organization or co-evolution, for example. However, from a methodological perspective and based on our experience, situational analysis allows the freedom to describe a situation occurring in a food system (for example) with complex adaptive systems concepts in mind. Properties of complex adaptive systems, either from Anderson and colleagues [7] or others such as Holland [37], could be included in a social worlds/arenas map to expand analysis to recognize how these properties manifest.

Complex adaptive systems principles of human and non-human agents and interconnections, as used by both Anderson and colleagues [7] and Clarke [12], are common for many research methods, including social network analysis and analysis based on actor-network theory. The researcher can identify the principle of self-organization in the situational maps where clusters are evident, based on behaviours that may result from coping with changes. The principle of emergence, which occurs when properties are distinct from individual agents but evolves from the actions of those agents, is evident through positional maps in Situational Analysis. The researcher does not attach positions in the data to a person or organization, but recognize the positions as they emerge from a higher-level understanding of the situation. Co-evolution is most evident when the analyst looks beyond the system boundaries in both past and current behaviours. For example, climate change is beyond the system boundaries but discussions of climate change have co-evolved with the growing local food movement. This may be more evident in studies with longitudinal data collection, to capture change over time. It is by observing the complex system as a whole in the situational maps, and understanding the history or evolution of that system, that we can consider how systems co-evolve and influence each other. There remains work in developing specific methods that capture and describe complexity science, particularly social complexity, and this study provides a launching point for further methodological development when studying complex adaptive systems.

## 5. Situational analysis and public health research

Situational analysis is well suited as a method to study context, as illustrated by the above example. It is particularly beneficial for the study of public health implementation research where the context of implementation has a strong influence on the outcomes of policies and programs. For example, implementing the new food security core public health program in the context of a growing local food movement resulted in changes for the way the food safety core program was delivered, with more lenience toward farmer's markets than in the past. We have found context to be important in our studies on evidence informed practice, the renewal of public health systems, and in examining the use of an equity lens in public health.

A main strength of situational analysis is the alignment of the method with concepts of complex adaptive systems. Research in many public health domains is limited when randomized controlled trials are the preferred method. Randomization is often impossible in public health programs because of equity issues, and context is stripped in most epidemiological research involving experimental and quasi-experimental designs. In complexity, context interacts with and thus is part of the intervention, so stripping it from study does not allow us to answer the important research questions. Recognizing public health as a complex adaptive system operating in real-world settings, and identifying an appropriate research method is essential to advancing public and population health outcomes.

Situational analysis does have weaknesses that are important to consider. As with grounded theory and many qualitative research methods, it is time consuming, particularly with large scale, multi-site studies. Coding data is only the beginning process, which is challenging to do well in large teams. Map making may benefit from multiple perspectives, but it is important for team members to have a depth of knowledge of the interviews when engaged in the map-making process. Using the social worlds/arenas map for knowledge translation purposes can be challenging as well. For this reason, when reporting to participants, Martin [13] included a separate diagram that was more closely aligned with a grounded theory diagram to explain the outcomes of her study because it was easier to communicate and understand. The positional maps, however, can be quite useful to guide the reader though discourses found in the situation.

## 6. Conclusion

As a research method, situational analysis is only beginning to be used in health related studies [38]. However, this method has the potential, along with Strauss' conditional matrix, to move public health knowledge beyond the individual and the basic social process, toward taken-for-granted structural conditions that can negatively affect the wellness of individuals and groups. This method can offer an alternative view on studies of intersectoral collaboration, social justice and the social determinants of health, all of which are inherent in the work of those engaged in public health and health promotion. Studies completed by the RePHSS team have demonstrated this method has great potential for work in areas of complexity, and will continue to seek the best way to engage complex adaptive systems concepts fully.

## References

[1] Di Ruggiero E, Frank J, Moloughney B (2004). Strengthen Canada's public health system now. Canadian J Public Health.

[2] Baker EL, Melton RJ, Stange PV (1994). Health reform and the health of the public: Forging community health partnerships. JAMA.

[3] Plianbangchang S, Organization WH (2007). Inauguration of University of Public Health, Address, 16 July 2007, Yangon Myanmar.

[4] MacDonald M, Hancock T, Wilson Strosher H (2007). Developing a research agenda on the implementaiton and impact of Core Public Health Functions in British Columbia.

[5] MacDonald M (2011). Developing a program of research for an applied public health chair in public health education and population intervention research. Can J Nursing Res.

[6] Tomm-Bonde L, Schreiber RS, Allan D (2013). Fading vision: knowledge translation in teh implementation of a public health policy intervention. Implementation Sci.

[7] Anderson RA, Crabtree BF, Steele DJ (2005). Case Study Research: The View From Complexity Science. Qualitative Health Research.

[8] Pettigrew AM (1990). Longitudinal field research on change: Theory and practice. Organization science.

[9] Poland B, Frohlich KL, Cargo M, Potvin L, McQueen D (2008). Context as a fundamental dimension of health promotion evaluation. Health promotion evaluation practices in the Americas: Values and research.

[10] Edwards N, Di Ruggiero E (2011). Exploring which context matters in the study of health inequities and their mitigation. Scandinavian J Public Health.

[11] Rychetnik L, Frommer M, Hawe P (2002). Criteria for evaluating evidence on public health interventions. J Epidemiology and Community Health.

[12] Clarke AE (2005). Situational Analysis: Grounded Theory After the Postmodern Turn.

[13] Martin W (2014). Food Gone Foul? Food Safety and Security Tensions.

[14] Hamm MW, Bellows AC (2003). Community Food Security and Nutrition Educators. J Nutri Edu & Behav.

[15] Thrift N (1999). The place of complexity. Theory, Culture & Society.

[16] Castellani B, Hafferty F (2009). Sociology and Complexity Science: A New Field of Inquiry.

[17] Capra F (1996). The Web of Life.

[18] Holland JH (1992). Complex Adaptive Systems. Daedalus.

[19] Zimmerman B, Lindberg C, Plsek P (2001). Edgeware: Insights from Complexity Science for Health Care Leaders.

[20] Tremblay M-C, Richard L (2011). Complexity: a potential paradigm for a health promotion discipline. Health Promotion Inter.

[21] Glaser B, Strauss AL (1967). The Discovery of Grounded Theory.

[22] Strauss AL (1978). A Social Worlds Perspective. Studies in Symbolic Interaction.

[23] Clarke AE, Maines DR (1991). Social worlds/arenas theory as organizational theory. Social organization and social process: Essays in honor of Anselm Strauss.

[24] Charmaz K, Morse JM (2009). Shifting the grounds: Constructivists grounded theory methods. Developing Grounded Theory: Teh Second Generation.

[25] Strauss AL (1993). Continual Permutations of Action.

[26] Healy K, Moody J (2014). Data Visualization in Sociology. Annual Rev Soci.

[27] Mead GH (1938). The Philosophy of the Act.

[28] Food Security Working Group (2006). Model core program paper: Food Security. BC Ministry of Health.

[29] Allen P (2008). Mining for justice in the food system: perceptions, practices, and possibilities. Agriculture Human Values.

[30] Province of British Columbia (2008). Bill 23 Public Health Act.

[31] (2012). Safe Foods for Canadians Act. Statutes of Canada 2012.

[32] Labonte R (1993). Health Promotion & Empowerment: Practice Frameworks.

[33] Lubell M (2007). Familiarity Breeds Trust: Collective Action in a Policy Domain. J Politics.

[34] Wittman H, Desmarais AA, Wiebe N (2011). Food Sovereignty in Canada.

[35] Blouin C, Lemay JF, Konforti L (2009). Local food systems and public policy: A review of the literature.

[36] Pimbert M (2010). Towards Food Sovereignty: Reclaiming autonomous food systems.

[37] Holland JH (1995). Hidden Order: How adaptation builds complexity.

[38] Schreiber RS, Martin W, Beck CT (2013). New directions in grounded theory. Routledge International Handbook of Qualitative Nursing Research.

